# Modelling long-term COVID-19 impacts on the U.S. workforce of 2029

**DOI:** 10.1371/journal.pone.0260797

**Published:** 2021-12-01

**Authors:** Shade T. Shutters

**Affiliations:** 1 School of Complex Adaptive Systems, Arizona State University, Tempe, Arizona, United States of America; 2 Global Climate Forum, Berlin, Germany; University of Gothenburg: Goteborgs Universitet, SWEDEN

## Abstract

While ensuring employment opportunities is critical for global progress and stability, workers are now subject to several disruptive trends, including automation, rapid changes in technology and skill requirements, and transitions to low-carbon energy production. Yet, these trends seem almost insignificant compared to labor impact of the COVID-19 pandemic. While much has been written about the pandemic’s short-term impacts, this study analyzes anticipated long-term impacts on the labor force of 2029 by comparing original 2029 labor projections to special COVID-adjusted projections recently published by the US Bureau of Labor Statistics. Results show that future demand for nearly every type of labor skill and knowledge will increase, while the nature of work shifts from physical to more cognitive activities. Of the nearly three million jobs projected to disappear by 2029 due to COVID, over 91% are among workers without a bachelor’s degree. Among workers with a degree demand shifts primarily from business-related degrees to computer and STEM degrees. Results further show that the socialness of labor, which is important for both innovation and productivity, increases in many more industries than it decreases. Finally, COVID will likely accelerate the adoption of teleworking and slightly decrease the rate of workforce automation. These impacts, combined with a shift to more cognitive worker activities, will likely impact the nature of workforce health and safety with less focus on physical injuries and more on illnesses related to sedentary lifestyles. Overall, results suggest that future workers will need to engage more often in training and skill acquisition, requiring life-long learning and skill maintenance strategies.

## Introduction

More than 3 million people globally migrate to cities each week, primarily in search of employment opportunities [1]. In a 2016 U.S. White House report on the future of cities, listed first among challenges facing urban residents was “finding and acquiring a good job, a quality education, and appropriate training” [2]. Similarly, in its 2016 report to Congress, the U.S. National Science Foundation designated “The Future of Work” as one of its 10 Big Ideas for Future Investment and a critical focus for future research. [3]. Thus, it is broadly acknowledged that improving human well-being is critically dependent on providing quality jobs and ensuring workers have appropriate skills.

Yet several ongoing trends have accelerated in recent years to cast uncertainty on the nature of work in the near future. Countries of the world are beginning clean energy transitions that will likely cause severe economic and labor disruptions in the short term [4,5], leading to possible high unemployment, increased migration, uncertainty in tax revenues, and social unrest [6,7]. Workers simultaneously face the specter of the so-called Fourth Industrial Revolution [8]–the use of robots and artificial intelligence to replace human labor. McKinsey Consulting estimates that 800 million jobs will disappear globally by 2030 [9], with unemployment potentially surpassing that experienced during the 2007–09 Great Recession [10]. At the same time, efficiency gains from automation is expected to add $16 trillion to the global economy by 2030 [11]. This means automation-related increases in global wealth will be shared by an ever-shrinking number of workers, intensifying inequality and accelerating regional fragility [12].

In the midst of these disruptive trends the COVID-19 pandemic (hereafter COVID) erupted in early 2020. The impacts of this unprecedented shock have been multiple and complex, including business closures, loss of child-care options, reduced consumer spending, and other impacts, none of which are isolated, but which interact in subtle ways to exacerbate the pandemic’s aggregate effects [13–17]. The pandemic has also exacerbated issues of labor inequality [18–20] and accelerated ongoing trends such as the adoption of telework [21,22]. Yet, most of what has been learned regarding impacts of the covid pandemic is focused on currently observable effects.

In this study I take advantage of a unique dataset recently published by the US Bureau of Labor Statistics (BLS) to isolate long term impacts on the skills and education composition of the US labor force. Because of the importance of person-to-person interaction in innovation and productivity, I also examine how the pandemic is likely to shape the socialness of future work, both in aggregate and within individual industries. Further, I determine whether the pandemic is likely to alter ongoing trends in automation and teleworking. Finally, I explore how COVID-induced changes in the nature of future work might affect the occupational health and safety of workers.

### Covid and the future labor force

Each year the BLS projects US national employment by industry and occupation 10 years into the future. This dataset, known as the National Employment Matrix, is typically published with a lag of 1–2 years. For instance, the 2029 projections were published in late 2020. Because of the unprecedented impact of the COVID pandemic on US labor, the BLS took the unusual step of preparing alternate scenarios of future employment projections, each assuming different long-term impacts of COVID on the structure of US labor. Two scenarios, termed medium and strong impact scenarios, were published in February 2021 modelling the impact of long-term effects of COVID, such as the transition to telework and movement from brick-and-mortar retail to eCommerce. These trends not only change labor demand in the affected industries but alter consumer behavior and supply chains in related industries.

A BLS analysis of these scenarios presents an excellent discussion of the industry and occupational shifts under the two COVID scenarios [23]. Among industry sectors, the authors show that accommodation and food services will suffer the largest decreases in employment due to COVID. Employment in arts, entertainment, recreation, retail trade, and construction also decreases significantly under the strong COVID scenario. On the other hand, the information sector as well as professional, scientific, and technical services are projected to increase employment in response to the pandemic. In terms of occupations, the report finds that food preparation/serving and sales jobs will experience the largest decreases in employment, while science, computer, and mathematical occupations will experience significant employment increases under the strong COVID scenario.

Firms, workers, and educators may be significantly affected by these changes in the future workforce. To better plan for those changes, it is important to consider factors beyond demand for individual occupations. Workers and economic planning agencies must be aware of changes in skills requirements while educational institutions must be ready to shift training options to meet the new needs of firms. Planners must also anticipate how trends in teleworking might impact infrastructure. Building on [23] this paper contributes to addresses these lingering issues by analyzing the long-term impacts of COVID on the future labor force in terms of skills composition, degree distributions, automation potential, and ability to telework.

In is important to clarify that this study does not compare employment patterns of today with employment patterns of 2029, which is covered in [23]. Instead, this study isolates the long-term impacts of COVID on the future workforce by comparing the 2029 strong and medium COVID employment scenarios to the original 2029 employment projections, which I refer to as the baseline scenario.

## Data and methods

### Employment projections

Employment projection data were published as three individual scenarios. For the baseline scenario I use the original 2019–2029 National Employment Matrix [24], published in late 2020. The medium and strong COVID impact scenarios were special data sets prepared by the BLS in 2021 [25]. These scenarios contain a mix of industries and occupations at multiple aggregation levels. Here, I include only ‘Line item’ occupations to avoid duplicate counting.

### Occupational skills and activities

To examine the COVID impact on future worker skills and activities I utilize the ONET dataset [26]. ONET decomposes US occupations into two types of attributes describing the nature of an occupation–elements, which have a continuous value, and intermediate work activities (IWAs), which have a present/absent flag. ONET element data are further grouped into element categories, which include skills, knowledge, abilities, and others. Some element categories, such as education, do not have a single value for an occupation but instead have a distribution. Such categories that do not have a single value per occupation are not included in this analysis. Throughout this analysis ONET elements collectively are referred to as skills.

Elements are further defined by a measurement scale, such as importance or level. Here I use values for importance (IM), except in the case of Work Context elements, which have no values for either importance or level. For those elements I instead use the context (CX) scale values.

Because ONET uses an expanded occupation code, those codes must be mapped to BLS occupation codes with a crosswalk file. Many ONET codes map to one BLS code. Thus, ONET values are mapped to BLS codes by simple averaging. The ONET to BLS occupation mapping must be created manually and is specific not only to the version of ONET but to the year and dataset of the BLS. Here the crosswalk is specific to ONET version 25.2 and the BLS’s 2019 National Employment Matrix.

As with elements, IWAs must be mapped to BLS occupations codes. Since there is no value to collapse by averaging, we adopt the method used in [27] to assign a present/absent indicator for each BLS occupation. If an activity is present in any one of the ONET occupations that map to a single BLS occupation, it is designated as present in the BLS occupation.

### College degrees and degree groups

To determine how the future distribution of college degrees changes under each COVID projection scenario I first create a probability distribution function for degrees by occupation derived from empirical data. This is obtained from the US Census Bureau’s 2019 American Community Survey (ACS) 1-year Public Use Microdata Sample (PUMS) [28]. These data comprise a survey of approximately 1% of the US population. Each record in this microdata represents a single individual but carries a weight factor which estimates of the total number of similar individuals in the individual’s geographic area.

While the microdata contains covers several hundred attributes of each survey respondent, I extract only occupation code (SOCP), 1st degree field of study (FOD1P), and person weight (PWGTP). If the field of degree is blank the respondent is designated as having no degree. Individuals without an occupation code are excluded from this study.

Like many US agencies, the Census Bureau maintains an idiosyncratic list of occupation codes that do not exactly match those used by the BLS. Thus, I construct a crosswalk to match many census occupation codes to one BLS occupation code. With this crosswalk, a degree probability distribution by census occupation is restated as a degree probability distribution by BLS occupation. This distribution of approximately 175 degrees is further aggregated into 37 degree groups and results for both degrees and degree groups are applied to occupational projections of each COVID scenario. COVID impact on degree demand is calculated both as the change in projected number of degrees in the national workforce and as a percentage of all workers.

### Industry “socialness”

I also calculate the “socialness” of industries under the two COVID projection scenarios. Socialness can significantly impact an industry’s projected productivity as well as an economy’s rate of innovation. I this study I calculate industry socialness as the number of social IWAs performed by workers of an industry divided by the industry’s total labor force. This measure of industry socialness as the number of social IWAs per worker. I adopt the determination of which IWAs are social from Painter et al [27] and apply these to each occupation in the industry’s employment projections. This allows comparison of industry socialness between the baseline projection and the two COVID impact scenarios. Results are calculated for both detailed industries (4-digit NAICS) and industry sectors (2-digit NAICS).

One exception is noted: Industry 453100 (Florists) was included in the baseline BLS projections but not in either COVID impact scenario and is thus excluded from the analysis.

### Job automation

To estimate job automation risk at the occupation level I use Frey & Osborne’s metric of automation potential by individual occupation code [29]. Those probabilities area applied to occupation-level employment under each COVID scenario to estimate projected changes in total US workforce susceptible to automation. These measures are also applied to employment in individual industries and industry sectors to understand how COVID may impact automation within industries.

### Teleworking estimates

To examine the impact of different scenarios on the future propensity to telework, I use a 2020 study from the U.S. National Bureau of Economic Research [30]. This study analyzes each occupation’s ONET characteristics to determine which occupations can be performed remotely [31]. I then apply those occupational designations to baseline and COVID impact projection scenarios and aggregate to both industry and national levels.

## Results

In each of the following analyses the baseline 2029 employment projections are compared to both the medium and strong COVID impact scenarios for 2029 and changes under each COVID scenario are presented.

### Impact on occupational elements and worker activities

COVID impacts on projected per capita importance of ONET elements is presented in Table 1. Elements decreasing the most in importance are largely associated with physical tasks, such as standing, walking, running, or moving objects, and with skills likely to be automated, such as interacting with the public. On the other hand, elements associated with data and data manipulation have the largest increases in per capita importance.

**Table 1:** Projected changes in importance of occupational elements.

Element Name (Element Type)	Baseline Value	Change: Medium Scenario	Change: Strong Scenario
Spend Time Standing (work contexts)	3.165	-0.0094	-0.0171
Performing for or Working Directly with the Public (work activities)	3.127	-0.0087	-0.0157
Food Production (knowledge)	0.776	-0.0087	-0.0143
Spend Time Walking and Running (work contexts)	2.644	-0.0083	-0.0142
Handling and Moving Objects (work activities)	3.407	-0.0065	-0.0126
Operations Analysis (skills)	1.278	0.0092	0.0161
Spend Time Sitting (work contexts)	2.756	0.0100	0.0168
Analyzing Data or Information (work activities)	3.037	0.0095	0.0172
Electronic Mail (work contexts)	3.423	0.0108	0.0179
Documenting/Recording Information (work activities)	3.002	0.0110	0.0195

Top and bottom five changes ranked by strong scenario impact are presented.

Results shown in Table 1 include all elements defined by ONET that have a single value for an occupation. However, this combines five categories of elements which may or may not be comparable despite sharing the same scale and range of values. Therefore, I present also elements having the largest positive and negative changes in each category in Table 2.

**Table 2:** Projected changes in importance of occupational elements by element type.

	Element Name	Baseline Value	Change: Medium Scenario	Change: Strong Scenario
**Abilities**				
	Gross body coordination	1.3155	-0.0066	-0.0114
	Extent flexibility	1.6202	-0.0062	-0.0113
	Trunk strength	2.0543	-0.0060	-0.0107
	Stamina	1.3748	-0.0063	-0.0107
	Static strength	1.6708	-0.0052	-0.0088
	Fluency of Ideas	2.5984	0.0043	0.0070
	Written comprehension	3.2791	0.0052	0.0087
	Inductive reasoning	3.0664	0.0051	0.0088
	Deductive reasoning	3.1591	0.0054	0.0094
	Written expression	3.0380	0.0059	0.0102
**Knowledge**				
	Food production	0.7762	-0.0087	-0.0143
	Sales and Marketing	2.1062	-0.0042	-0.0085
	Customer and Personal Service	3.8963	-0.0020	-0.0033
	Foreign language	0.9591	-0.0006	-0.0009
	Production and Processing	1.9748	0.0011	0.0013
	Clerical	2.8582	0.0057	0.0094
	Design	1.4404	0.0063	0.0101
	Education and Training	3.0480	0.0054	0.0107
	Engineering and Technology	1.5628	0.0073	0.0116
	Computers and Electronics	2.9597	0.0083	0.0130
**Skills**				
	Service orientation	2.8406	0.0001	0.0000
	Negotiation	2.4695	0.0009	0.0015
	Operation and Control	1.3868	0.0012	0.0017
	Coordination	2.9693	0.0016	0.0029
	Management of Personnel Resources	2.3828	0.0017	0.0030
	Programming	0.5603	0.0063	0.0099
	Systems evaluation	2.2575	0.0059	0.0104
	Reading comprehension	3.2232	0.0064	0.0108
	Science	0.8209	0.0083	0.0151
	Operations analysis	1.2779	0.0092	0.0161
**Work activities**				
	Performing for or Working Directly with the Public	3.1267	-0.0087	-0.0157
	Handling and Moving Objects	3.4065	-0.0065	-0.0126
	Selling or Influencing Others	2.3626	-0.0027	-0.0060
	Performing general physical activities	2.7609	-0.0034	-0.0057
	Controlling Machines and Processes	2.3626	-0.0011	-0.0029
	Organizing, Planning, and Prioritizing Work	4.0718	0.0082	0.0149
	Interpreting the Meaning of Information for Others	2.6571	0.0085	0.0149
	Making Decisions and Solving Problems	3.7335	0.0084	0.0151
	Analyzing Data or Information	3.0367	0.0095	0.0172
	Documenting/recording information	3.0016	0.0110	0.0195
**Work Contexts**				
	Spend Time Standing	3.1649	-0.0094	-0.0171
	Spend Time Walking and Running	2.6436	-0.0083	-0.0142
	Deal With External Customers	3.4570	-0.0052	-0.0102
	Deal With Unpleasant or Angry People	3.0366	-0.0048	-0.0092
	Spend Time Making Repetitive Motions	3.0257	-0.0039	-0.0082
	Consequence of Error	2.7326	0.0036	0.0071
	Time Pressure	3.6313	0.0041	0.0075
	Letters and Memos	2.8995	0.0060	0.0104
	Spend Time Sitting	2.7561	0.0100	0.0168
	Electronic Mail	3.4233	0.0108	0.0179

Top and bottom five changes ranked by strong scenario impact are presented for each element type.

Not one element in the skills category exhibits a decrease in importance. Similarly, all but four of the elements in the Knowledge category increase in importance under the COVID scenarios.

In addition to ONET elements, ONET also decomposes occupations into a set of over 200 Intermediate Work Activities (IWAs) which are tabulated as either present or absent. Table 3 presents the impact of COVID on the fraction of total workers performing each IWA.

**Table 3:** Projected changes in fraction of workforce engaged in Intermediate Work Activities (IWAs).

Intermediate Work Activity (IWA)	Baseline Value	Change: Medium Scenario	Change: Strong Scenario
Clean tools, equipment, facilities, or work areas	0.3925	-0.0042	-0.0080
Provide general assistance to others, such as customers, patrons, or motorists	0.1489	-0.0043	-0.0079
Respond to customer problems or inquiries	0.2358	-0.0039	-0.0079
Execute financial transactions	0.2508	-0.0037	-0.0070
Stock supplies or products	0.1317	-0.0030	-0.0060
Maintain health or medical records	0.1204	0.0009	0.0021
Design computer or information systems or applications	0.0666	0.0013	0.0022
Develop operational or technical procedures or standards	0.1136	0.0020	0.0031
Maintain operational records	0.4832	0.0018	0.0035
Maintain current knowledge in area of expertise	0.2740	0.0019	0.0036

Top and bottom five changes ranked by strong scenario impact are presented.

Under both the strong and medium scenarios of COVID impact, demand for skills shifts to areas concentrated in computers and health care. These include skills in programming, chemistry, biology, and several related scientific and medical fields.

The IWA with the highest increase in demand is maintenance of current knowledge and expertise, suggesting that future workers will be required to engage more in life-long learning and continuous skill acquisition [32].

### Impact on demand for college degrees

While the previous results demonstrate the increased importance of skills and knowledge, here we examine how those changes are manifest across college degrees. The largest COVID impacts on 173 different degrees are presented both as total degrees (Table 4) and as percent of total workers (Table 5). Results reveal the largest decreases in demand are concentrated in business degrees, while the largest increases are in STEM degrees, particularly those related to computer science and engineering. Viewing results instead as a percent of workforce (Table 5) changes the story significantly. Only three of 173 degrees are projected to decrease as a percent of workforce–hospitality management, cosmetology and culinary arts, and drama and theater arts.

**Table 4:** Projected changes in total workers by degree.

Degree Name	Baseline Value	Change: Medium Scenario	Change: Strong Scenario
N/A (less than bachelor’s degree)	110,686,780	-1,193,600	-2,628,411
Business Management and Administration	3,024,906	-6,593	-23,796
General Business	2,161,253	-6,281	-20,188
Psychology	2,363,213	-5,777	-18,024
Accounting	1,822,978	-5,401	-15,252
Communications	1,207,943	-4,314	-12,698
Chemistry	530,655	3,523	2,302
Computer and Information Systems	427,943	5,927	5,796
Computer Engineering	329,390	5,403	6,143
Electrical Engineering	959,986	7,847	6,632
Computer Science	1,265,810	20,043	22,500

Top and bottom five changes ranked by strong scenario impact are presented.

**Table 5:** Projected changes in percent of workforce holding each degree (shown as a percentage).

Degree Name	Baseline Value	Change: Medium Scenario	Change: Strong Scenario
N/A (less than bachelor’s degree)	65.5593	-0.2201	-0.4144
Hospitality Management	0.1428	-0.0005	-0.0011
Cosmetology Services and Culinary Arts	0.0340	-0.0003	-0.0005
Drama and Theater Arts	0.1319	-0.0001	-0.0004
Military Technologies	0.0019	0.0000	0.0000
Visual and Performing Arts	0.0399	0.0001	0.0000
Electrical Engineering	0.5686	0.0090	0.0141
Business Management and Administration	1.7916	0.0095	0.0176
Biology	1.0560	0.0120	0.0196
Nursing	1.4999	0.0095	0.0236
Computer Science	0.7497	0.0176	0.0269

Top and bottom five changes ranked by strong scenario impact are presented.

Detailed degree data are further aggregated into 37 degree groups and COVID impacts area show both in terms of total degrees (Table 6) and percent of workforce (Table 7). At this level of aggregation several degree groups decrease in total quantity, with business degrees having the largest drop. However, only the degree group personal and culinary services decreases as a percent of workforce.

**Table 6:** Projected changes in total workers by degree group.

Degree Group Name	Baseline Value	Change: Medium Scenario	Change: Strong Scenario
N/A (Less Than Bachelor’s Degree)	110,686,780	-1,193,600	-2,628,411
Business, Management, Marketing, and Related Support Services	10,352,520	-25,588	-85,966
Visual and Performing Arts	2,090,532	-8,470	-27,292
Education	4,580,233	-10,163	-27,205
Social Sciences	3,652,598	-6,909	-24,750
Communication, Journalism, and Related Programs	2,147,882	-6,354	-20,757
Mathematics and Statistics	684,532	2,587	1,230
Physical Sciences	1,496,544	6,075	1,676
Biological and Biomedical Sciences	2,682,433	13,686	5,612
Engineering	3,976,403	17,668	6,289
Computer and Information Sciences and Support Services	2,001,828	30,432	32,399

Top and bottom five changes ranked by strong scenario impact are presented.

**Table 7:** Projected changes in percent of workforce holding each degree group (shown as a percentage).

Degree Group Name	Baseline Value	Change: Medium Scenario	Change: Strong Scenario
N/A (Less Than Bachelor’s Degree)	65.5593	-0.2201	-0.4144
Personal and Culinary Services	0.0340	-0.0003	-0.0005
Military Science, Leadership and Operational Art	0.0019	0.0000	0.0000
Mechanic and repair technologies/technicians and precision production	0.0097	0.0001	0.0001
Science Technologies/Technicians	0.0095	0.0001	0.0002
Construction Trades	0.0546	0.0001	0.0002
Education	2.7129	0.0143	0.0320
Health Professions and Related Programs	2.6892	0.0179	0.0387
Computer and Information Sciences and Support Services	1.1857	0.0271	0.0407
Engineering	2.3552	0.0282	0.0458
Business, Management, Marketing, and Related Support Services	6.1318	0.0308	0.0576

Top and bottom five changes ranked by strong scenario impact are presented.

### Impact on industry and sector workforce socialness

COVID impacts on industry socialness are calculated for both 2-digit industry sectors and 4-digit detailed industries. Among 2-digit sectors (Table 8), only 3 industry sectors decrease in socialness under the strong COVID scenario, while all others increase. Among 4-digit industries (Table 9) several industries show decreases in socialness. Interestingly, one of these is scientific research and development (5417), which displays a marked decrease in socialness even though the sector of which it is part, professional, scientific and technical services (54), exhibits one of the largest *increases* in sociality among sectors. This is indicative of a large degree of heterogeneity in attributes of the individual industries that comprise an industry sector.

**Table 8:** Projected changes in social IWAs per worker by 2-digit industry sector.

Industry Sector (Code)	Baseline Value	Change: Medium Scenario	Change: Strong Scenario
Agriculture, forestry, fishing and hunting (11)	2.2485	-0.0106	-0.0080
Construction (23)	3.1154	-0.0061	-0.0077
Accommodation and food services (72)	1.8663	-0.0169	-0.0063
Utilities (22)	4.3561	-0.0009	0.0003
Educational services; state, local, and private (61)	3.6670	0.0011	0.0015
Finance and insurance (52)	3.8202	0.0059	0.0141
Retail trade (44–45)	1.5623	0.0154	0.0206
Professional, scientific, and technical services (54)	5.5047	0.0089	0.0214
Arts, entertainment, and recreation (71)	2.5853	0.0122	0.0260
Information (51)	4.9138	0.0242	0.0316

Top and bottom five changes ranked by strong scenario impact are presented. Government sectors are excluded.

**Table 9:** Projected changes in social IWAs per worker– 4-digit industries.

Industry Name (code)	Baseline Value	Change: Medium Scenario	Change: Strong Scenario
Scientific research and development services (5417)	6.2893	-0.0890	-0.0884
Manufacturing and reproducing magnetic and optical media (3346)	3.6782	0.0311	-0.0829
Agents and managers for artists, athletes, entertainers, etc. (7114)	2.1779	0.0007	-0.0639
Inland water transportation (4832)	2.4888	0.0158	-0.0459
Vending machine operators (4542)	2.0129	-0.0447	-0.0418
Drinking places, alcoholic beverages (7224)	1.7682	0.0053	0.0587
Scenic and sightseeing transportation, other (4879)	1.7619	0.0714	0.0714
RV (recreational vehicle) parks and recreational camps (7212)	2.9168	0.0443	0.0793
Forestry (1131–2)	5.7802	0.0962	0.0962
Scenic and sightseeing transportation, land (4871)	1.8021	0.0816	0.1027

Top and bottom five changes ranked by strong scenario impact are presented.

### Impact on workforce automation

Using Frey & Osborne’s [29] occupation level metric of automation potential, I calculate the fraction of jobs in each 2-digit industry sector (Table 10) and 4–digit industries (Table 11) that are susceptible to automation. Results reveal that among 2-digit sectors, only construction (23) and transportation/warehouse (48–49) have an increased probability of automation under the strong COVID scenario.

**Table 10:** Projected change in fraction of jobs susceptible to automation by 2-digit industry sector.

Industry Sector (Code)	Baseline Value	Change: Medium Scenario	Change: Strong Scenario
Retail trade (44–45)	0.7487	-0.0045	-0.0065
Arts, entertainment, and recreation (71)	0.5697	-0.0029	-0.0061
Professional, scientific, and technical services (54)	0.3770	-0.0032	-0.0050
Information (51)	0.3517	-0.0025	-0.0043
Other services (except public administration) (81)	0.4876	-0.0016	-0.0032
Accommodation and food services (72)	0.8677	0.0000	-0.0006
Mining, quarrying, and oil and gas extraction (21)	0.6147	-0.0002	-0.0005
Agriculture, forestry, fishing and hunting (11)	0.7332	0.0004	0.0000
Construction (23)	0.6069	0.0005	0.0006
Transportation and warehousing (48–49)	0.7032	0.0006	0.0009

Top and bottom five changes ranked by strong scenario impact are presented.

**Table 11:** Projected change in fraction of jobs susceptible to automation by 4-digit industry.

Industry Name (Code)	Baseline Value	Change: Medium Scenario	Change: Strong Scenario
Rooming and boarding houses, dormitories, and workers’ camps (7213)	0.6342	-0.0067	-0.0160
Scenic and sightseeing transportation, other (4879)	0.6106	-0.0124	-0.0124
Health and personal care stores (4461)	0.6883	-0.0069	-0.0100
Clothing stores (4481)	0.8182	-0.0087	-0.0094
Promoters of performing arts, sports, and similar events (7113)	0.6492	-0.0017	-0.0090
Alumina and aluminum production and processing (3313)	0.6779	0.0000	0.0017
Urban transit systems (4851)	0.6617	0.0000	0.0024
Logging (1133)	0.7480	0.0029	0.0024
Inland water transportation (4832)	0.5123	0.0009	0.0044
Vending machine operators (4542)	0.7806	0.0053	0.0076

Top and bottom five changes ranked by strong scenario impact are presented.

### Impact on teleworking

Analyzing COVID’s impact on the probability that jobs can be performed remotely we find that results are highly skewed toward higher probability of teleworking (Table 12). Among 2-digit industry sectors only construction (23) displayed a decrease in fraction of jobs that can be performed remotely. This suggests that long term impacts of COVID include an acceleration of telework adoption by workers.

**Table 12:** Projected changes in fraction of jobs that can be performed remotely by 2-digit industry sector.

Industry Sector (Code)	Baseline Value	Change: Medium Scenario	Change: Strong Scenario
Construction (23)	0.1789	-0.0002	-0.0004
Agriculture, forestry, fishing and hunting (11)	0.3750	0.0001	0.0000
Accommodation and food services (72)	0.0271	0.0001	0.0002
Mining, quarrying, and oil and gas extraction (21)	0.2331	0.0002	0.0003
Educational services; state, local, and private (61)	0.8124	0.0006	0.0007
Other services (except public administration) (81)	0.3952	0.0013	0.0029
Arts, entertainment, and recreation (71)	0.2922	0.0024	0.0032
Finance and insurance (52)	0.7956	0.0009	0.0034
Information (51)	0.7534	0.0032	0.0037
Retail trade (44–45)	0.1417	0.0036	0.0046

Top and bottom five changes ranked by strong scenario impact are presented.

While several industries at the 4-digit industry do exhibit decreases in potential for telework, the magnitude of those changes is relatively small compared to the industries expected to increase adoption of teleworking (Table 13).

**Table 13:** Projected changes in fraction of jobs that can be performed remotely by 4-digit industry.

Industry Name (Code)	Baseline Value	Change: Medium Scenario	Change: Strong Scenario
Promoters of performing arts, sports, and similar events (7113)	0.4694	0.0020	-0.0073
Charter bus industry (4855)	0.1251	0.0011	-0.0019
Coal mining (2121)	0.0541	0.0000	-0.0017
Other support activities for transportation (4889)	0.2447	-0.0016	-0.0016
Fruit and vegetable preserving and specialty food manufacturing (3114)	0.1072	-0.0015	-0.0014
Rooming and boarding houses, dormitories, and workers’ camps (7213)	0.1896	0.0030	0.0103
RV (recreational vehicle) parks and recreational camps (7212)	0.3375	0.0068	0.0110
Scenic and sightseeing transportation, other (4879)	0.0856	0.0111	0.0112
Scenic and sightseeing transportation, land (4871)	0.1776	0.0090	0.0115
Travel arrangement and reservation services (5615)	0.4585	0.0070	0.0128

Top and bottom five changes ranked by strong scenario impact are presented.

## Discussion

The following discussion covers several topics related to this study. For clarity, the study’s key findings are summarized in Table 14.

**Table 14:** Summary of key long-term COVID impacts on future labor.

• the nature of work will shift from physical tasks to more cognitive tasks
• workers will spend more time maintaining skills or learning new ones, especially computer skills
• workers without a college degree will have much fewer job opportunities
• demand for college degrees will shift from business fields to STEM and computer fields
• automation will accelerate in construction and transportation/warehousing sectors, but slow in most others
• teleworking will grow in most industries
• worker safety will focus less on physical injuries and more on illnesses related to sedentary lifestyles

### The nature of future work: Skill requirements and job activities

Results generally suggest that the pandemic will induce future work to be less physical and more cognitive in nature. For instance, worker abilities with the largest increases in importance (Table 2) include reasoning, comprehension, and fluency of ideas, which are all cognitive abilities. On the other hand, worker abilities decreasing most in importance are physical attributes of workers, such as stamina, strength, and flexibility.

Worker skills in particular increase in importance. In fact, not one ONET skill decreases in importance under either COVID scenario. This suggests that skill acquisition by workers will be central to future employment and further highlights the key role that education, training, and life-long learning will play in preparing and maintaining the future workforce.

A similar pattern is exhibited by COVID impacts on intermediate work activities (Table 3). Future workers will perform fewer routine and repetitive tasks, such as cleaning tools or stocking supplies, and perform more cognitive tasks, such as developing procedures, maintaining records, and designing computer applications. While many IWAs diminishing in prevalence are physical activities that might be automated by robots, others are more cognitive in nature, such as executing financial transactions, that are susceptible to automation through computerization.

These results suggest the pandemic has accelerated the key role that education will play in preparing the workers in the future. It is perhaps telling that the work activity with the highest projected increase is Maintaining current knowledge in area of expertise (Table 3). Thus, a key task of future workers will be the activity of continuously upkeeping skills and knowledge.

### Education and learning activities

The shift in future labor attributes from physical to cognitive work is further reflected in the forecasted importance of college degrees. Of the nearly 2.9 million jobs expected to disappear under the strong COVID scenario, 91% are jobs that do not currently require a college degree (Table 4). Thus, job-seekers with a high school education or below will bear the brunt of lost opportunities under future COVID scenarios.

In terms of the quantity of college degrees in the workforce, COVID the largest decreases will be among business and related degrees while the largest increases are in computer-related degrees (Table 4). When aggregated to degree groups, the number of business-related degrees in the workforce will decrease by nearly 86,000, while computer-related degrees will increase by over 32,000 (Table 6). Other STEM degree groups, such as engineering, biological and physical sciences, and statistics also increase in number, despite there being almost 3 million less workers in the workforce.

Analyzing aggregate numbers of degrees gives only a partial picture of COVID’s impact on the post-secondary education landscape. It is also important to analyze the *relative* distribution of those degrees and examining the percentage of the workforce holding each type of degree offers another perspective of COVID’s impact on worker education. For instance, despite having the largest drops in aggregate numbers of degrees, business degrees nevertheless increase as a percentage of the workforce (Tables 5 and 7).

This occurs because, despite the decrease in quantities of certain degrees, the decrease in workers with no degree overwhelms all other categories. Thus, even though nearly 2.9 million jobs disappear under the strong COVID scenario, the percent of the workforce with a degree increases by over 4%, which positively impacts nearly every degree category. In fact, only three of 173 detailed degrees are projected to decrease as a percent of workforce–hospitality management, cosmetology and culinary arts, and drama and theater arts. And while computer science continues to show the largest increase in relative terms, degrees such as nursing and biology also increase significantly (Table 5). Aggregated to degree groups, only Personal and Culinary Service degrees decrease as a percentage of workforce.

It is important to note that the underlying data sources do not address other types of post-secondary education, such as 2-year degrees, high value certificates, microcredentials, and other worker training options. Thus, it is difficult to discern the importance of these credentials with the given data. However, it is likely that these non-traditional pathways for education, which were identified as critical to the future workforce [32], will only increase in importance as the need to continually maintain and update skills accelerates under COVID.

### Industry socialness: Impacts on innovation and productivity

When ONET work activities are classified as either social or non-social and applied to employment projections, a measure of labor socialness can be calculated by industry. Social IWAs are defined as those likely to require face-to-face interaction between employees. Painter et al [27] recently demonstrated that the spatial density of social ONET elements is positively correlated with patenting output of cities and that the number of social IWAs per worker in an industry is superlinearly related to the industry’s per worker GDP. Thus, socialness is positively correlated with both innovation and productivity. Here I calculate industry socialness as the number of social IWAs per industry worker.

Results show that, among 2-digit industry sectors (Table 8), socialness increases most in Arts and Recreation (71). Socialness also increase substantially under the strong COVID scenario in the Finance (52), Information (51), and Professional/Scientific Services (54) sectors. Thus, it is plausible that the long-term impacts of COVID may include increased productivity and rates of innovation in these sectors.

Only three sectors exhibit a decrease in socialness under the strong COVID scenario. These are Agriculture (11), Construction (23), and Accommodation/Food Services (72), possibly reflecting a growing trend in mechanization and automation in these sectors.

This result indicates that the nature of work is likely to shift in ways that better facilitate workplace innovation. Thus, industry leaders and policy makers might seek to exploit this increased workforce socialness by implementing policies and strategies that foster innovation and reward creativity.

### Trends in workforce automation and teleworking

Workforce automation, which has been ongoing for years, accelerated during the first months of the COVID pandemic [32]. This study suggests that this acceleration in automation may only be a short-term response and that COVID will slow the rate of automation on longer time scales. Among 2-digit sectors (Table 10), only construction (23) and transportation/warehouse (48–49) showed an increased probability of automation under the strong COVID scenario. Though the effect is negligible, it is unclear why this would be, and further research is warranted into the long-term impacts of COVID on trends in workforce automation.

A further long-term impact of COVID is an increase in nearly every industry of the amount of work performed remotely (Table 12). Among 2-digit industry sectors only construction (23) displayed a decrease in the fraction of jobs that can be performed remotely.

At the 4-digit industry level results are mixed and several industries do exhibit decreases in potential for telework. Yet, there is no immediately obvious trend among those that increase and decrease at the 4-digit level. For instance, charter buses (4855), other transportation activities (4889), and sightseeing transportation (4871, 4879) are all part of the same industry sector, yet charter buses and other transportation activities both decrease in teleworking potential while sightseeing transportation increases (Table 13).

Therefore, we can only conclude from this study that COVID will induce a broad increase in teleworking across industry sectors and across the workforce in aggregate. Still this may be informative for planning infrastructure investments for the future workforce, as this broad trend will likely increase the need for services such as broad band internet [33,34], while decreasing the demand for other services [35,36].

It should also be noted that teleworking changes the nature of worker interactions. Because those interactions are central to the previously discussed worker socialness that is linked to innovation and productivity, accelerated adoption of teleworking may mitigate projected impacts on workplace innovation. Further research is thus warranted to understand how interpersonal interactions and communication change in telework environments.

### Occupational health and safety considerations

The COVID-induced shift away from physical activities to more cognitive activities in the future workforce may have significant implications for workplace health and safety. Because workplace injuries are often associated with physical tasks [37], it is reasonable to expect that workplace injuries will decrease under the strong COVID scenario. At the same time, reduced physical activity can induce new health issues related to a sedentary lifestyle, including mental health and stress-related illnesses [38]. Indeed, the ONET element “Spend Time Standing” decreases more than any other Work Context element, while “Spend Time Sitting” exhibits the second largest increase among Work Contexts. Thus, occupational health and safety professionals and policy makers should be aware of these potential shifts in workplace health risks.

At the same time, the anticipated increase in telecommuting means that more workers will perform tasks remotely in often isolated and unsupervised environments without typical workplace safeguards [39,40]. This creates additional health and safety considerations [41] as well as numerous legal and liability questions and thus should be a target for further research.

### Caveats and opportunities

It is critical to understand that the conclusions of this study are based on long-term employment forecasts and, even though these forecasts are the product of sophisticated and well-vetted methodology, such forecasts are inherently uncertain. It is also important to realize that the changes in projected skills and education due to COVID are aggregate economic impacts and do not consider changes that may occur in the job requirements of individual occupations over the next 10 years. In other words, while this analysis uses the skills and knowledge requirements of today’s jobs those job requirements may change by 2029 –both because of long-term impacts of COVID and simply because of ongoing trends in technology, training, and innovation. Such changes in occupational requirements happen frequently and are especially rapid in STEM fields [42].

This issue applies equally to the potential for automation to radically change the activities and requirements of individual occupations. Factors used in this study for susceptibility to automation and potential for telecommuting apply to today’s jobs but will almost surely change in the future. For instance, advances in connectivity and changes in corporate culture will influence the prevalence of telecommuting while unforeseen advances in technology will likely accelerate automation. Both trends can also be significantly affected by policies, such as tax regimes or labor laws.

Given that such changes in occupational structure will occur in parallel to changes in demand for various occupations, this analysis may nevertheless help regional policy makers reprioritize planning for industry growth and educational provision. Overall, this analysis indicates that COVID will increase the need for post-secondary educational options. While this study points to increased demand in traditional degrees it is more likely that a suite of innovative options will be needed to prepare the workforce of the future for economies shaped by the long-term impacts of COVID.

Finally, readers should be aware of shortcomings of the underlying data and issues that may arise from linking disparate datasets. Consider the ONET data used in this study to assign skills, knowledge, and activities to individual occupations. The ONET data provides occupation specific values for attributes of the occupation “Accountant”. However, an accountant in New York City may have significantly different skills requirements than an accountant in a small rural village, while an accountant in a large financial consulting firm may have very different activities and knowledge requirements than an accountant in a small family-owned restaurant. Such differences are ignored as they are simply beyond the scope of such a dataset. Thus, geographical and economic context must be considered when using the results of this study to inform policy.
